# Recurrent Eccrine Acrospiroma of the Parotid Region: A Case Report

**DOI:** 10.31729/jnma.5702

**Published:** 2021-12-31

**Authors:** Vikas Gupta, Utkal Priyadarshi Mishra, Ganakalyan Behera

**Affiliations:** 1 Department of ENT-Head and Neck Surgery, All India Institute of Medical Sciences, Bhopal, 462020, India

**Keywords:** *acrospiroma*, *neoplasms*, *skin*

## Abstract

Eccrine acrospiroma is a benign tumor of skin and adnexa arising from eccrine sweat gland epithelium. It is usually solitary, slow growing tumor commonly affecting extremities. Rarely it affects head and neck region, and extremely rare in parotid region. Females are affected more often. Treatment of choice is wide local excision with adequate skin margins. Although benign this tumor is very notorious for recurrence after inadequate resection. We describe here a case of young male patient with recurrent eccrine acrospiroma over parotid region which was managed by wide local excision with primary repair with excellent results.

## INTRODUCTION

Eccrine acrospiroma is a benign adnexal tumor arising from distal part of excretory ductal epithelium of eccrine sweat gland. It is also known as clear cell/nodular hidradenoma, eccrine hidradenoma, porosyringoma, clear cell papillary carcinoma, clear cell myoepithelioma.^1,2^ It is usually a solitary and slow growing tumor involving skin of eyelid, face, neck & extremities. Malignant acrospiroma are not uncommon & have propensity for lymph node metastasis. Treatment consists of wide local excision alone with adequate skin margins for benign tumor & neck dissection with post op radiotherapy for its malignant counterparts. We report a case of recurrent eccrine acrospiroma located on parotid region in a young male.

## CASE REPORT

A 25-year-old male presented to Otorhinolaryngology outpatient department with complaint of a swelling over right infra auricular region for last eighteen months. He underwent surgery in the form of local excision one year back, at another center. Histopathology of excised mass was consistent with eccrine acrospiroma (nodular hidrad enoma). Swelling reappeared at the same site within four months of surgery. Prior to presentation at our center, he noticed a rapid increase in size of the swelling over a period of two months.

**Figure 1:**
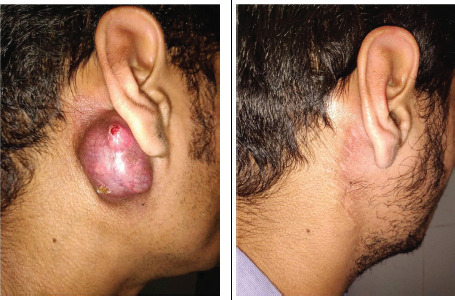
A) Preoperative clinical picture, B) Follow up after 1 year showing well healed surgical site.

Local examination revealed a 4x4cm, solitary, firm to cystic, nodular swelling over right parotid area extending from right infra auricular area to mastoid tip. The swelling was bluish to pinkish in color and was found adhered to skin as well as underlying deep structures with areas of surface ulceration and serous discharge. No palpable cervical lymphadenopathy was noted. Fine needle aspiration cytology from the lesion was reported as benign adnexal tumor (Figure 1).

**Figure 2:**
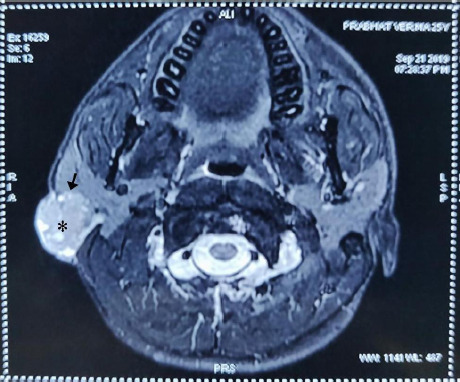
T2 weighted Contrast enhanced MRI showing lesion (asterisk) & loss of fat planes with parotid tissue (black arrow).

Contrast enhanced MRI revealed a large lobulated soft tissue mass in right facial soft tissue with heterogeneous signal on T2 and low signal on T1 found to be involving the superficial lobe of right parotid gland with no extension into the deep lobe and involving overlying subcutaneous fat and bulge in the overlying skin. Mass measured 3.5x2.2x2.7cm approximately. Multiple enlarged lymph nodes of size 10x15mm are seen in right parotid region and infra parotid region (Figure 2).

**Figure 3:**
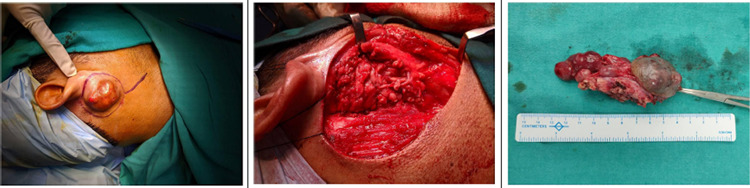
A. Incision Markings. B. Skin defect after excision of tumor. C. Excised specimen.

A wide local excision of the lesion along with selective level II lymph node dissection under general anesthesia was performed. Circumferential skin incision was made around swelling taking 1cm margin all around. Incision was extended inferiorly along neck for around 5cms (Figure 3A). Intraoperatively, swelling was centered in the area between mastoid process and posterior margin of mandible and firmly adhered to the underlying deep fascia covering sternocleidomastoid muscle and posterior part of the parotid gland. Multiple enlarged lymph nodes of size 2x2cm to 1x1cm were found over right level II region. Lesion was separated from underlying sternocleidomastoid muscle along with deep fascia. Inferior part of the tumor was adhered to the parotid tissue close to marginal mandibular nerve. Facial nerve trunk was identified and marginal mandibular branch was carefully separated from tumor and preserved. Around 2cm margin of the normal parotid tissue was resected along with the lesion, preserving the inferior branches of the facial nerve. Inferiorly the lesion was found adhered to level II enlarged nodes which were dissected and removed in continuity with the lesion. An oriented specimen is sent for HPR (Figure 3 A, B and C).

Wide undermining of wound edges done and skin defect was repaired primarily in layers with vacuum suction drain in situ. Patient had paresis of right marginal mandibular branch of facial nerve in immediate post op period which subsequently improved over a period of 2 weeks. Post op histopathology revealed a diagnosis of eccrine acrospiroma without any features of a malignant change. All the excised lymph nodes were of reactive nature on histopathology. Patient is under close follow up and is disease free till one year follow up with a slightly visible surgical scar (Figure 1B).

## DISCUSSION

Eccrine acrospiroma is a benign tumor of the skin originating from sweat gland ductal epithelium. Term “Eccrine acrospiroma" was first introduced by Johnson and Helwig in 1969 where “Spiroma" stands for adenoma of sweat glands and “acro" indicates the top most or end.^3^ It is a very slow growing tumor affecting mostly middle-aged females. It can occur in any part of body but commonly found in face (30%), scalp (10%),trunk (14%), foot (15%) and hand (5%).^4^ Malignant transformation is rare but can arise de novo or in long standing cases. Malignant counterpart behaves very aggressively with rapid increase in size and metastasis to regional lymph nodes.^5^

Acrospiroma usually presents as a solitary flesh colored solid or cystic nodule of 1 to 2cm size with occasional skin ulceration and leakage of serous fluid from it. Skin over the swelling is usually pink or purple in color and thinned out. Very rarely they become painful and violaceous.

Histopathology reveals an encapsulated tumor with solid nest of cells with focal ductal differentiation sharply demarcated from the dermis. It contains both solid and cystic components in varying proportions. Solid nest of cells consists of two types of cell population; predominant cell types are a polyhedral cell, which shows faintly eosinophilic granular cytoplasm and round to oval nucleus. The other cell type is a pale or clear cell with eccentric nucleus and no identifiable cytoplasm which contain glycogen and periodic acid-Schiff — positive, diastase-resistant material.^6,7^ They express cytokeratin, epithelial membrane antigen and p53.^8^

Differential diagnosis includes hemangioma, poroma, infected sebaceous cyst, metastatic lymphadenopathy.

Management of choice for eccrine acrospiroma is wide local excision with at least 1cm skin margin. Anything less than that will lead to recurrence. Recurrence after excision have been reported by Johnson and Helwig in 38 out of 319 cases.^3^ Certain histological features may identify a high risk of recurrence such as dermal lymphatic invasion, nerve sheath involvement, deep structural infiltration, positive resected margins, and extracapsular lymph node extension.^9^

In our case, recurrence may be attributed to inadequate local excision during the previous surgery or simply to the nature of the disease. We also addressed this recurrent tumor with local excision; but with adequate margins and a selective lymph node dissection for occult metastasis. Malignancy was ruled out by histopathology which also ruled out any adjuvant treatment in the form of radiation. Accordingly, patient is on close follow up only.

Mainstay of treatment for malignant acrospiroma is wide local excision with lymph node dissection with post-operative radiotherapy. Role of radiotherapy is well established at a dose of 50-70Gy in preventing recurrence following surgical excision. Chemotherapy is usually reserved for recurrent or metastatic malignant acrospiroma. In one study capecitabine in a dose of 1500 mg/m2 has shown good results in metastatic hidradenocarcinoma.^9^

Next generation sequencing has not shown any identifiable mutations that would mandate targeted therapy in malignant acrospiroma.^9^
